# Vectorial wall shear stress calculations in vessel structures using 4D PC-MRI

**DOI:** 10.1186/1532-429X-14-S1-W5

**Published:** 2012-02-01

**Authors:** Wouter V Potters, Pim van Ooij, Ed vanBavel, Aart Nederveen

**Affiliations:** 1 Radiology, Academic Medical Centre, Amsterdam, Netherlands; 2 Biomedical Engineering and Physics, Academic Medical Centre, Amsterdam, Netherlands

## Summary

We propose a fully automated method for calculating vectorial wall shear stress (WSS) in-vivo based on 4D PC-MRI data.

## Background

Wall shear stress (WSS) is the tangential force of flowing blood on the vessel wall. WSS directly influences remodeling of the vessel wall.

## Methods

Velocity data were corrected for aliasing and phase offsets and subsequently filtered using a median filter. Inward unit normal vectors were determined on the wall, after which a coordinate transformation was performed for each point at the wall such that the normal vector coincided with the z-axis of the transformed coordinate system. Velocities at fixed points along the normal were calculated using natural 3D interpolation in the original data. Any perpendicular velocity components were removed as only tangential velocity components contribute to the viscous forces at the wall. Smoothing splines were then fitted to the x- and y- velocity components along the inward unit normals. The x- and y-derivatives at the vessel wall were derived analytically and multiplied with the viscosity, which resulted in the WSS. Finally, all WSS vectors were transformed back to the original coordinate system.

This method was validated using a synthetic dataset of a rigid straight tube (diameter 6mm) with parabolic flow, in which the theoretical WSS could be derived analytically (Poiseuille). Effects of resolution, segmentation errors and noise were assessed using this phantom data. Secondly the algorithm was tested in in-vivo PC-MRI data. In vivo PC-MRI of the common carotid artery was performed on a 3T MRI system (Philips Healthcare, Best, The Netherlands) using a dedicated 8 channel carotid coil, 5 heart phases, FOV 80x80x12 mm, isotropic voxel size 0.4 mm (non-interpolated), sense factor 2, Venc of 30 (ap), 30 (rl) and 70 (fh) cm/s, scantime 10 minutes.

## Results

**Figure 1:**
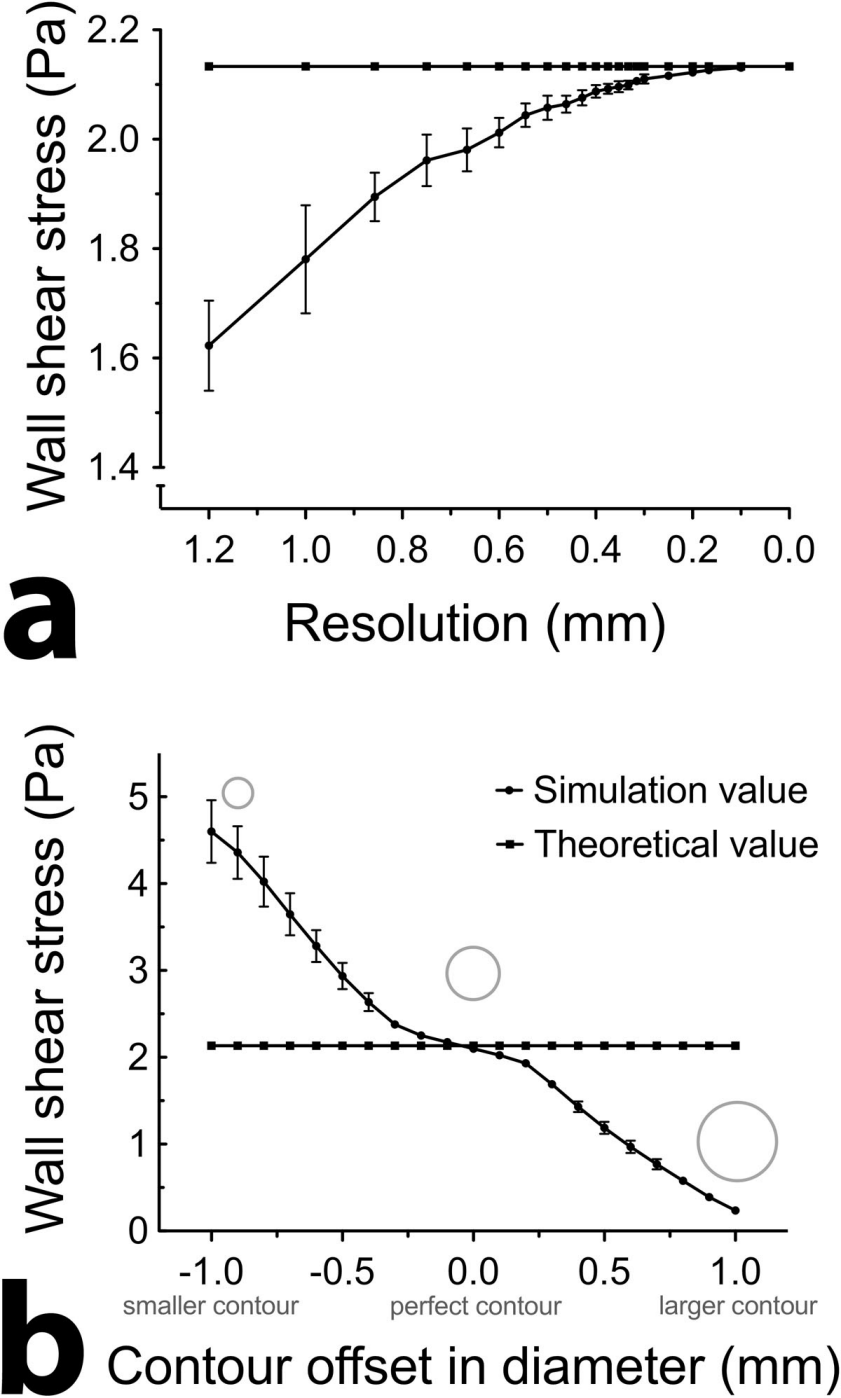
a) Effect of increasing resolution on the calculated WSS in phantom data. WSS values converge to the theoretical value (calculated through Poiseuille flow). The standard deviation is caused by the discretization at different resolutions. b) Effect of segmentation errors on the calculated WSS in phantom data. The circles illustrate the used segmentation. Constriction of the segmentation leads to overestimation of the WSS, where expansion leads to underestimation of the WSS.

The phantom study reveals that increasing the resolution will result in improved approximations of the theoretical WSS (figure 1a). Additionally the standard deviation (SD) of WSS declines with increasing resolution (figure 1a). Secondly, the effect of false segmentations (change in diameter) on the calculated WSS was substantial (figure 1b). Addition of Gaussian white noise had a limited impact on the SD in WSS calculations (5% increase for noise with 4 cm/s variance, 15% increase for noise with 16 cm/s variance).

**Figure 2:**
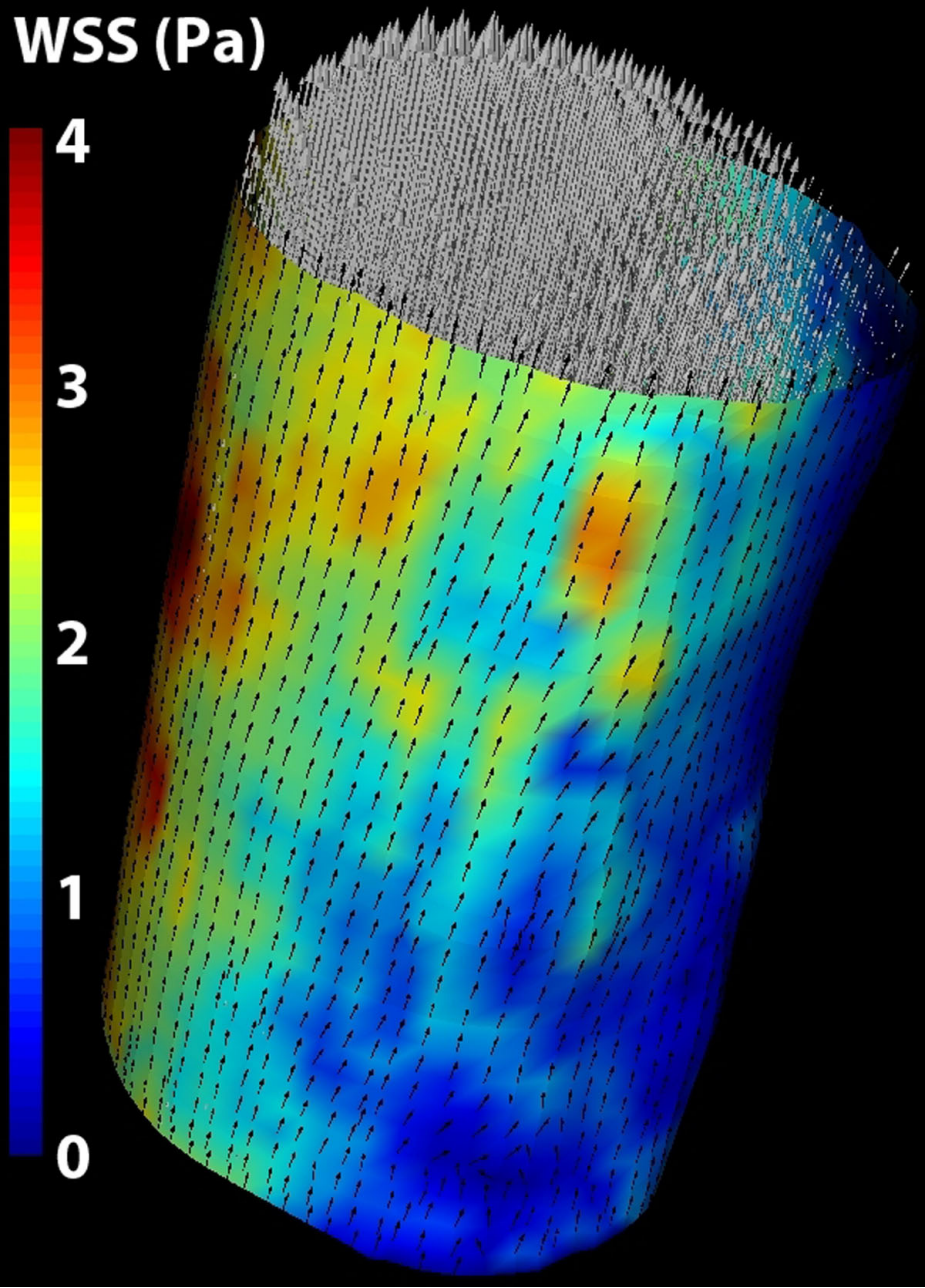
Wall shear stress calculated in an in-vivo measurement of the common carotid artery. Velocity vectors are shown as white quivers, normalized WSS vectors are shown as black arrows on the wall. Colors visualize the WSS magnitude.

The in-vivo WSS shows good visual resemblance with the measured flow profile. The calculated WSS values are within the physiological range (figure 2).

## Conclusions

This work presents a new method to determine WSS in-vivo independent of assumptions on the flow profile. Results suggest that a resolution of at least 0.6 mm should be used for a 6mm diameter vessel to calculate the WSS within 10% of theoretical values. High-resolution images are needed to avoid segmentation errors. Further validation of the algorithm in-vivo is planned.

## Funding

None.

